# No evidence for attenuated stress-induced extrastriatal dopamine signaling in psychotic disorder

**DOI:** 10.1038/tp.2015.37

**Published:** 2015-04-14

**Authors:** D Hernaus, D Collip, Z Kasanova, O Winz, A Heinzel, T van Amelsvoort, S M Shali, J Booij, Y Rong, M Piel, J Pruessner, F M Mottaghy, I Myin-Germeys

**Affiliations:** 1 Department of Psychiatry and Psychology, South Limburg Mental Health Research and Teaching Network, EURON, School for Mental Health and NeuroScience MHeNS Maastricht University, Maastricht, The Netherlands; 2 Department of Nuclear Medicine, University Hospital RWTH Aachen University, Aachen, Germany; 3 Department of Nuclear Medicine, Academic Medical Center, Amsterdam, The Netherlands; 4 Institute of Nuclear Chemistry, Johannes Gutenberg University, Mainz, Germany; 5 Department of Psychiatry, Douglas Mental Health Institute, McGill University, Montreal, QC, Canada; 6 Department of Nuclear Medicine, Maastricht University Hospital, Maastricht, The Netherlands

## Abstract

Stress is an important risk factor in the etiology of psychotic disorder. Preclinical work has shown that stress primarily increases dopamine (DA) transmission in the frontal cortex. Given that DA-mediated hypofrontality is hypothesized to be a cardinal feature of psychotic disorder, stress-related extrastriatal DA release may be altered in psychotic disorder. Here we quantified for the first time stress-induced extrastriatal DA release and the spatial extent of extrastriatal DA release in individuals with non-affective psychotic disorder (NAPD). Twelve healthy volunteers (HV) and 12 matched drug-free NAPD patients underwent a single infusion [^18^F]fallypride positron emission tomography scan during which they completed the control and stress condition of the Montreal Imaging Stress Task. HV and NAPD did not differ in stress-induced [^18^F]fallypride displacement and the spatial extent of stress-induced [^18^F]fallypride displacement in medial prefrontal cortex (mPFC) and temporal cortex (TC). In the whole sample, the spatial extent of stress-induced radioligand displacement in right ventro-mPFC, but not dorso-mPFC or TC, was positively associated with task-induced subjective stress. Psychotic symptoms during the scan or negative, positive and general subscales of the Positive and Negative Syndrome Scale were not associated with stress-induced [^18^F]fallypride displacement nor the spatial extent of stress-induced [^18^F]fallypride displacement in NAPD. Our results do not offer evidence for altered stress-induced extrastriatal DA signaling in NAPD, nor altered functional relevance. The implications of these findings for the role of the DA system in NAPD and stress processing are discussed.

## Introduction

In the last decade, significant progress has been made in understanding the role of the dopamine (DA) system in the human stress response.^1, 2, 3^ Evidence has emerged showing that, at least in part, the stress response is facilitated by DA release in the striatum^1, 2, 4^ and prefrontal cortex (PFC).^5, 6^ Dopaminergic (DAergic) involvement in the stress response is particularly relevant for psychiatric disorders such as psychotic disorder,^7^ as evidence suggests that stress has an important role in the onset of psychotic symptoms^8, 9^ and DAergic abnormalities are a hallmark feature of psychotic disorder.^10^ Investigating stress-related DAergic activity in the context of psychotic disorder could thus provide new insights into the pathogenesis of the disorder.

Stress-induced DAergic activity in humans has been studied *in vivo* with positron emission tomography (PET), hinging on competition between radioligand binding and endogenous DA release.^11^ In these studies, DA release was assessed during a psychosocial evaluation paradigm^2^ (for metabolic stress, see Adler *et al.*^12^, Brunelin *et al.*^13^). Although psychosocial stress in healthy volunteers (HV) produced modest and variable changes in striatal DA release,^1, 2, 4, 14^ the same stressor reliably increased DA release in the (associative) striatum of individuals across the psychosis continuum.^1, 2, 4^ Importantly, this suggests that the putative association between stress and psychotic disorder may be moderated by the DA system.

Preclinical work, however, has revealed that short-lived stressors consistently and primarily increase DAergic activity in the PFC analog of the rodent.^15, 16^ Moreover, selective destruction of frontal DA neurons increases stress-related DA transmission in mesolimbic regions,^16, 17^ hinting at a key regulatory role for PFC DA transmission in the stress response. Because DA-mediated hypofrontality is hypothesized to be a cardinal feature of psychotic disorder,^18, 19^ this preclinical work indirectly suggests that the well-documented link between stress and psychotic disorder^20, 21^ may be underlain by cortical DA function. More specifically, decreased cortical DA function may constitute a neurochemical feature of vulnerability to psychotic disorder and underlie increased behavioral stress sensitivity.^21^

In the only two human studies currently available, psychosocial stress in HV increased medial PFC (mPFC) DA release^6^ and increased the area (that is, spatial extent) of mPFC DA release^5^ assessed with high-affinity D_2/3_ binding ligand [^18^F]fallypride.^22^ In an add-on sample of first-degree relatives of patients with psychotic disorder, Lataster *et al.*^3^ showed that the spatial extent of stress-induced mPFC DA release decreased as a function of increased subjective stress. Although this latter finding hints at stress-related DA-mediated hypofrontality in the psychosis continuum, investigating stress-induced PFC DAergic activity in established psychotic disorder could further elucidate the role of this mechanism in the pathogenesis of the illness. To these aims, we investigated the effect of psychosocial stress on extrastriatal DA signaling in a sample of HV and medication-free individuals with a diagnosis of non-affective psychotic disorder (NAPD) using [^18^F]fallypride PET.

However, measuring extrastriatal DAergic activity remains methodologically challenging; the density of extrastriatal D_2_ receptors is 2–8% compared with the striatum.^23^ Radioligands with suboptimal affinity and selectivity to investigate DAergic activity in extrastriatal areas may yield low signal-to-noise ratio, thus limiting quantification.^11, 24^ Although [^18^F]fallypride has been used to quantify DA release in cortical regions due to its high affinity and specificity, the effects of amphetamine on extrastriatal DA release quantified using [^18^F]fallypride have not been uniformly consistent.^25, 26, 27, 28, 29^ While this has been attributed to the radioligand's inherent signal-to-noise ratio,^26, 28^ within-subject variation introduced by two-day scanning protocols, with control and experimental scan on separate days, may also constitute a source of measurement error, particularly in the context of subtle changes in neurotransmitter activity. To minimize within-subject variation, we utilized a validated single infusion [^18^F]fallypride paradigm, which circumvents subtraction and yields a model fit approach.^3, 30, 31^

In the current study, medication-free NAPD and HV underwent a well-validated experimental psychosocial stress paradigm, the Montreal Imaging Stress Task (MIST).^2^ All the participants completed a MIST control and stress condition in a single [^18^F]fallypride session. Subjective stress responses, psychotic symptoms and plasma cortisol levels were assessed throughout each condition. Conform previous work, we first investigated stress-induced [^18^F]fallypride displacement and the spatial extent of stress-induced [^18^F]fallypride displacement in mPFC,^5, 6^ after which we explored other extrastriatal regions. It was expected that both outcome parameters of DA signaling would be positively associated with the subjective stress response in HV. Consistent with the notion of DA-mediated hypofrontality, we expected that NAPD would show less stress-induced extrastriatal [^18^F]fallypride displacement and a decrease in the spatial extent of stress-induced extrastriatal [^18^F]fallypride displacement, compared with HV.

## Materials and methods

### Sample

The sample consisted of 12 HV (unrelated to Lataster *et al.*^5^) and 12 NAPD matched on age, gender and education (Table 1). All NAPD were diagnosed with a non-affective psychotic disorder (Supplementary Table 1). Four included NAPD were antipsychotics naive. Except for one NAPD, the remaining group was treated with antipsychotics for <2 years. At the time of scanning, NAPD were off antipsychotics for at least 1 year (Table 1), were not exposed to mood stabilizers, were off antidepressants (total *n*=5) for longer than 1 year and did not take benzodiazepines on the day of the scan (Supplementary Table 1). NAPD showed relatively low acute psychotic symptom scores (Table 1), but did not meet the criteria for remission according to the Positive and Negative Syndrome Scale (PANSS) criteria (less than a score of 3 on all relevant items according to van Os *et al.*^32^). HV were matched to NAPD with a past of minimal illicit drug use (Table 1).

Participants were recruited through regional and national media and, additionally, NAPD were recruited through local mental health services. The RWTH Aachen University ethics committee approved the study. PET approval was granted by the national authority for radiation protection in humans in Germany (Bundesamt für Strahlenschutz, BfS). Written informed consent was obtained before participation. Inclusion criteria independent of group: (i) age 18–60 years (ii) able to provide informed consent. Exclusion criteria independent of group: (i) current/past use of illicit drugs according to the Composite International Diagnostic Interview (World Health Organization, 1990) (lifetime: >15 times cannabis, >5 times other drugs; illicit drug use in the past year), (ii) foreign bodies precluding a magnetic resonance imaging (MRI) scan, (iii) neurological disease, (iv) pregnancy. NAPD-specific inclusion criterion: diagnosis of non-affective psychotic disorder according to the Diagnostic and Statistical Manual of Mental Disorders (DSM-IV) criteria. HV-specific exclusion criteria: lifetime history of psychiatric illness according to DSM-IV criteria and lifetime neuroleptic use. On the day of scanning, a urine screening was performed to exclude current drug use and pregnancy.

### Psychosocial stress paradigm

**Figure 1:**
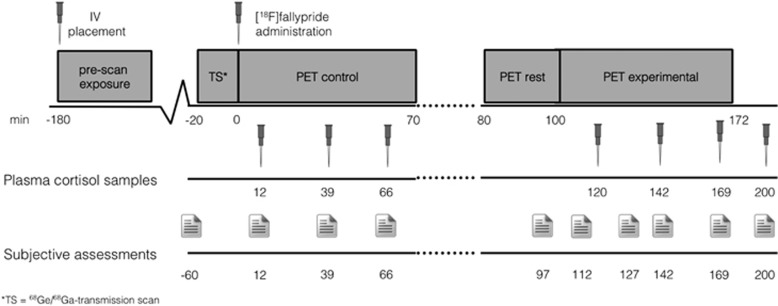
Graphical overview of the single infusion design. Following the transmission scan, the radioligand was injected after which participants always performed the control block of the MIST for 70 min. After a 10-min break, participants were repositioned using the scanner coordinate system and reference skin marks. At 100 min post injection, participants performed the MIST experimental condition for 70 min. Plasma cortisol samples were collected in intervals ranging from 22 to 54 min. MIST, Montreal Imaging Stress Task; PET, positron emission tomography.

Psychosocial stress was induced using the MIST.^2^ The MIST is a mental arithmetic task with an evaluative psychosocial component and has been prescribed in detail before.^1, 2, 5, 33^ Psychosocial feedback during the MIST was scripted. All participants were exposed to identical feedback by an investigator who was previously unknown to them. Time and difficulty were automatically adjusted during the experimental condition using a computer algorithm preventing users from exceeding 60–70% correct answers. The MIST training version was practised for 15 min at least 2 h before scan. Participants completed 10 6-min blocks of MIST control and experimental version. Control and experimental sessions were separated by a break (Figure 1).

### Behavioral and physiological assessments

PANSS positive, negative and general symptoms^34^ were assessed by a trained researcher before the scan. Subjective stress and psychotic symptoms were briefly assessed pre-scan (*n*=1), during each PET part (*n*=8) and post scan (*n*=1) (Figure 1). Subjective stress responses were assessed using seven-point Likert Scale items: ‘I feel relaxed' (reversed), ‘I feel judged' and ‘I do not live up to expectations', on the basis of previous work (*α*=0.69).^5, 33^ Psychotic symptoms (positive only) during the scan were assessed using the following items: ‘I hear voices', ‘I see things' and ‘I feel suspicious' (*α*=0.7). Plasma cortisol samples were also collected throughout each PET part (*n*=6) and post scan (*n*=1; Figure 1). Plasma cortisol levels were determined using a radio immunoassay.^35^

### Image acquisition and analyses

#### MRI scan

T1-weighted MRI scans were acquired on a 1.5T Philips (Philips Medical Systems, Herrsching, Germany) machine with TE=4.59 ms, TR=30 ms, matrix dimensions=256 × 256, slice thickness=2 mm, slice number=176. During the data acquisition phase, this scanner was replaced by a Siemens 3T scanner (Siemens Healthcare, Munich, Germany). Remaining scans (37.5%) were collected using the Magnetization Prepared Rapid Acquisition Gradient-Echo sequence, with TE=2.52 ms, TR=1900 ms, matrix dimensions=256 × 256, slice thickness=1 mm, slice number=176. A similar proportion of HV (5/12) and NAPD (4/12) MRI scans were obtained on the second machine.

#### Radioligand preparation

The radiosynthesis of [^18^F]fallypride was a high-yield modification of the synthesis method for [^18^F]desmethoxyfallypride, described in detail previously.^36, 37^

#### PET acquisition

All PET measurements were performed in the supine position in a quiet environment. Head position was fixed using a vacuum plastic mould to limit the head movement.^38^ The scans were performed in three-dimensional mode on a Siemens ECAT EXACT HR+ scanner (Siemens-CTY, Knoxville, TN, USA). Sixty-three slices of 2.425 mm slice thickness (pixel size=2 mm × 2 mm) were reconstructed per time frame by filtered back projection (Hamm filter) after Fourier rebinning into two-dimensional sinograms. Data sets were corrected for random coincidences, scatter radiation and attenuation (10 min ^68^Ge/^68^Ga-transmission scan). The image matrix was 128 × 128. The PET data were smoothed (4 mm FWHM), realigned, co-registered (transformation matrix based on first 10 realigned frames) (PMOD v3.1 (PMOD Technologies, Zurich, Switzerland)) and normalized (SPM 8, Wellcome Trust, London, UK). For every participant, an attenuation-corrected average image of the first 15 min was created. These frames were chosen because of their minimal amount of movement and subsequent high signal-to-noise ratio.^39^ The remaining frames were realigned to the 15-min mean image using squared difference sum (dissimilarity function) and trilinear interpolation as rigid matching settings in PMOD v3.1 and inspected frame by frame. To quantify the remaining discrepancy between mean frame and other frames, individual data sets X, Y, Z and pitch, roll, yaw parameters were exported from SPM 8 (realign option with trilinear interpolation). HV and NAPD did not differ in movement parameters (data upon request) and total sample movement parameters were low (X, Y, Z movement all <5 mm and pitch, roll, yaw all <5°).

Data were collected in two segments, a control and experimental part, in a single session with single bolus administration.^3, 33^ The PET acquisition protocol is visualized in Figure 1. Dynamic frames were collected every 60 s for the first 6 min, after which they were collected every 120 s for the remainder of the emission scan, in accordance with previous work.^3^ Break frames typically consisted of frame 39–42 and were discarded before preprocessing.

#### PET analysis

Time–activity curves were obtained for the cerebellum (reference region) and temporal and frontal regions. Two masks were created: one containing cerebellum only and another containing all regions (results section). Regions were based on Brodmann definitions, identical to previous work.^5, 33^ Using the Automated Anatomical Labeling mask provided by PMOD v3.1, hippocampus and amygdala were located for all participants. Using the PMOD v3.1 crop and tailor functions, hippocampus and amygdala were drawn and inspected slice by slice to ensure mask coverage. All masks were custom-tailored to the individual's MRI, transferred to co-registered PET data in PMOD v3.1 and visually inspected for fit by two independent raters. Given that striatal and extrastriatal regions differ in time to reach pseudo equilibrium, stress-induced [^18^F]fallypride displacement in striatal regions was not investigated; these values could not be reliably investigated with the current design, which was optimized to detect extrastriatal DA signaling.^31^

PET data were analyzed using a modified simplified reference tissue model,^40^ in accordance with previous work.^3, 30, 31, 33, 41, 42, 43, 44^ Stress-induced [^18^F]fallypride displacement, reflecting DA release, was quantified using time–activity curve plots and receptor kinetic parameters. The statistically significant change in radioligand displacement was calculated for every region of interest (ROI) as the *Z*-value of γ (γ/std(γ)).^33, 41^ Here, γ is considered an additional time-varying parameter in the simplified reference tissue model estimating the amplitude of ligand displacement at start of the experimental condition in a single scan session (based on the assumption that changes in competition between DA release and radioligand competition are reflected in the estimation of γ^31^). Given that this design does not assume a physiological steady state, it is suitable to investigate time-varying changes in DA concentrations. The *Z*-value of γ as a proxy of stimulus-induced changes in DA release is highly correlated with BP_ND (_binding potential relative to non-displaceable radioligand)^33, 41^ and has been validated using [^18^F]fallypride.^43^

γ was calculated over an exponential decay function *h*(*t*)=exp(−*τ*(*t*−*T*)), where *t*=measurement time, *T*=time of experimental condition initiation and *τ* controls the rate at which activation effects die away (dissipation rate, set to *τ*=0.03 min^−1^),^3, 31, 43^ yielding a γ variate estimation interval peaking at 11 min after experimental condition onset, with the peak dissipating to 10% in 69 min.

Because previous work has demonstrated that psychological paradigms not only affect the intensity (amount) of DA release, but also the area affected,^3, 33, 45^ the spatial extent of [^18^F]fallypride displacement was calculated as the percentage of voxels in an ROI showing significant radioligand displacement (quantified as γ) after correction (*p*(/number of total voxels)). This approach requires that voxel *T*-values in a given ROI are homogenously distributed for groups of interest (HV, NAPD); this assumption was tested by calculating the decrease in number of active voxels (that is, significant γ values) when increasing the *T*-value by 1 (tested for multiple *T-*values) in all ROIs and comparing this between groups (data upon request). High correlations (up to *r*=0.87) between ROI ligand displacement and the spatial extent of ligand displacement (in voxels) were observed, suggesting that the area of DA release increases with DA release.

### Analyses

Similar to previously published work investigating stress-induced [^18^F]fallypride displacement^6^ and the spatial extent stress-induced [^18^F]fallypride displacement,^3^ the total sample consisted of 12 matched HV and NAPD. *A priori* power analyses indicated a power of 0.82 to detect a group difference which is comparable to previous work using [^18^F]fallypride.^3^

Multilevel regression models with subject as the within level were applied to investigate increases in subjective stress and (positive) psychotic symptoms from control to experimental condition. Difference scores (stress-control condition) for subjective stress/symptoms were calculated for follow-up analyses. The area under the curve^46^ was calculated for plasma cortisol levels (nmol l^−1^). The area under the curve or nmol l^−1^ cortisol difference values were used for all cortisol analyses. Regions with mean BP_ND_ <0.5 in HV were not taken into account to prevent a low signal-to-noise ratio.

To replicate previous findings, we first investigated stress-induced mPFC [^18^F]fallypride displacement and the spatial extent of stress-induced mPFC [^18^F]fallypride displacement in HV. This was followed by an attempt to discover additional extrastriatal regions involved in stress processing in HV (Table 2 for all identified regions). For these purposes, *t*-tests (spatial extent/radioligand displacement >0) were performed. The same procedure was repeated for NAPD; no additional regions were identified in NAPD. Next, group differences (HV vs NAPD) in stress-induced radioligand displacement and its spatial extent were investigated in regions showing significant stress-induced radioligand displacement (using analysis of variance).

Follow-up analyses were performed using stress-induced increases in subjective stress/psychotic symptoms, symptom scores on PANSS subscales (positive, negative, general)^34^ and the amount of years off antipsychotics (day of scan−last day of antipsychotics use/365) as outcome variables. The *α* was set to the conventional threshold of *P*=0.05. Given the matched nature of the samples, covariates were not included in group comparisons. When analyzing single groups, age and gender were entered as nuisance covariates.

## Results

### Demographics, behavioral and physiological assessments

Groups did not differ on demographic variables (Table 1; all not significant). Recreational illicit drug use ceased long before the scan and no included participants reported current drug use (years since last use (*M*=17.83, s.d.=7.52)). Antipsychotics naive NAPD (*n*=4) and antipsychotics-free (currently non-medicated >1 year) participants did not differ in their PANSS score on the positive subscale (*t*(1,23)=0.25, *P*=0.81). Subjective stress during the scan increased from control to experimental condition (*b*=0.63, *z*(188)=6.07, *P*<0.0001), regardless of group (*b*=−0.24, *z*(1188)=−1.14, *P*=0.26). NAPD increased in positive psychotic symptoms from control to stress condition (*b*=0.21, *z*(95)=2.79, *P*=0.005). Subjective stress in the whole sample (*b*=−1.24, *z*(116)=−7.93, *P*<0.001) and positive psychotic symptoms in NAPD (*b*=−0.26, *z*(58)=−2.21, *P*=0.03) significantly decreased following a debriefing session 15 min after the scan finished. Cortisol (nmol l^−1^) decreased as a function of time in HV (*b*=−0.34, *t*(64)=−2.87, *P*=004), but not in NAPD (*b*=−0.02, *z*(66)=−0.11, *P*=0.91).

There were no differences in area under the curve cortisol between conditions (*t*(18)=1.65, *P*=0.12), nor were there group differences (*b*=474.42, *t*(1,9)=0.21, *P*=0.83) in area under the curve cortisol difference scores or an association with subjective stress (*b*=671.38, *t*(18)=0.43, *P*=0.67).

### Stress-induced [^18^F]fallypride displacement: main effects and group differences

The average HV BP_ND_ calculated over the whole paradigm using the simplified reference tissue model^40^ in the mPFC (*M*=0.51, s.d.=0.2), temporal cortex (TC; *M*=0.63, s.d.=0.16), hippocampus (*M*=1.56, s.d.=0.88), parahippocampal gyrus (*M*=0.66, s.d.=0.18) and amygdala (*M*=4.13, s.d.=1.56) was higher than 0.5 These regions were therefore included in the mask. No additional regions with mean BP_ND_ >0.5 were identified in NAPD.

In the mPFC and TC, a significant stress-induced increase in radioligand displacement and the spatial extent of radioligand displacement could be observed in HV and NAPD separately (*P*<0.05), but not in the hippocampus, parahippocampal gyrus or amygdala (*P*>0.05). No group differences in stress-induced radioligand displacement were observed in *a priori* selected ROI, the mPFC (Table 2), nor when looking at the dorso-mPFC (*b*=−0.05, *t*(1,23)=−0.12, *P*=0.91) or ventro-mPFC (*b*=−0.09, *t*(1,23)=−0.23, *P*=0.82) subregions separately. Moreover, no group differences in stress-induced radioligand displacement were observed in the TC (Table 2).

**Figure 2:**
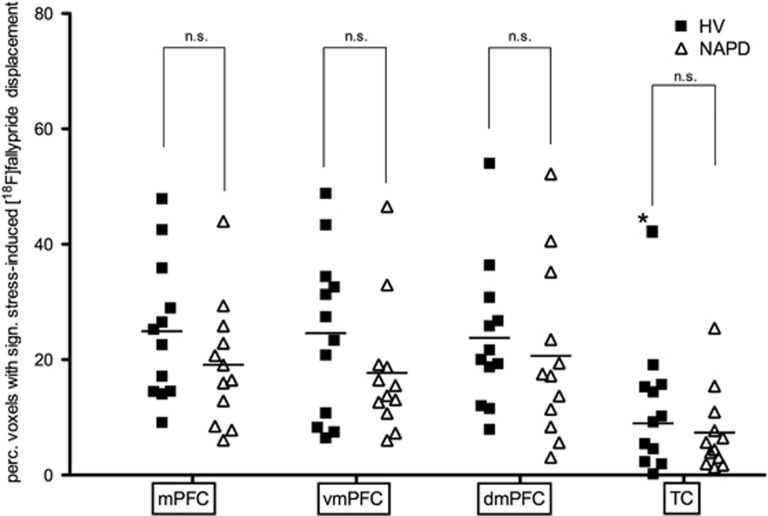
Group averages for the spatial extent of stress-induced [^18^F]fallypride displacement. NAPD did not significantly differ from HV in the spatial extent of stress-induced [^18^F]fallypride displacement in any (sub)region. Ventro-mPFC (vmPFC) and dorso-mPFC (dmPFC) are mPFC subregions. *, outlier (Cook's distance >4 per *n*), removed from mean. Not significant (NS) at *P*=0.05. HV, healthy volunteer; mPFC, medial prefrontal cortex; NAPD, non-affective psychotic disorder; TC, temporal cortex.

**Figure 3:**
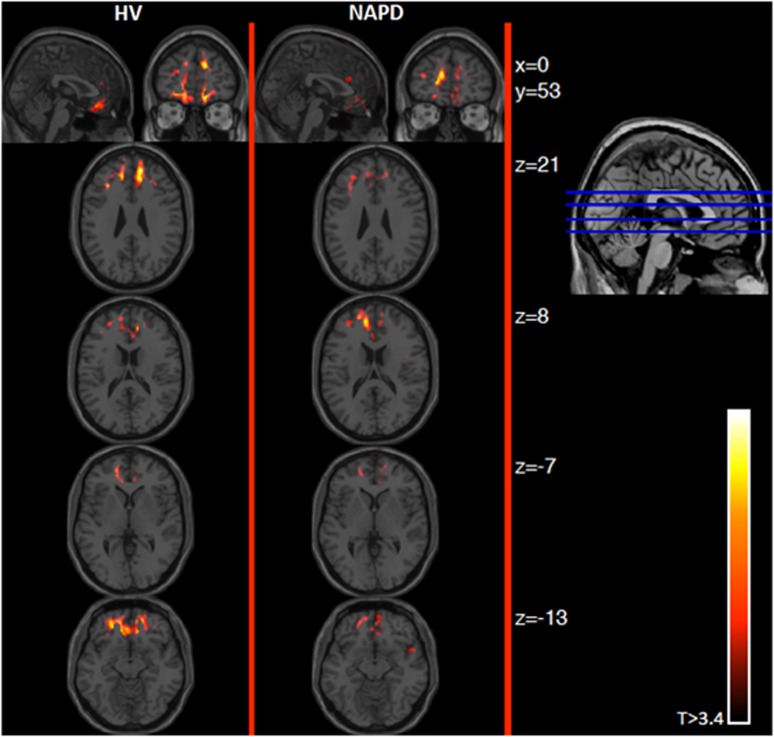
Parametric maps showing stress-induced [^18^F]fallypride displacement in mPFC. Graphical representation showing stress-induced [^18^F]fallypride in HV and NAPD in coronal (top row, left images), sagittal (top row, right images) and axial view (columns). Coronal image and Montreal Neurological Insititute (MNI) z coordinates on the right depict the axial slice position. Starting position (top) was *x*=0, *y*=53, *z*=21 (MNI). Mean t-maps per group show the stress-induced [^18^F]fallypride displacement throughout the mPFC. Individual t-maps were generated using displacement parameter γ (*t*=γ/sd(γ)) and were averaged across all participants per group. Images are thresholded at 3.4 for visualization purposes. HV, healthy volunteer; mPFC, medial prefrontal cortex; NAPD, non-affective psychotic disorder.

Similarly, no group differences were observed in the spatial extent of stress-induced radioligand displacement in the mPFC (Table 2), dorso-mPFC (*b*=−3.11, *t*(1,23)=−0.55, *P*=0.59), ventro-mPFC (*b*=−6.86, *t*(1,23)=−1.3, *P*=0.21) or TC (Table 2) (Figures 2 and 3).

### Stress-induced [^18^F]fallypride displacement: follow-up analyses

**Figure 4:**
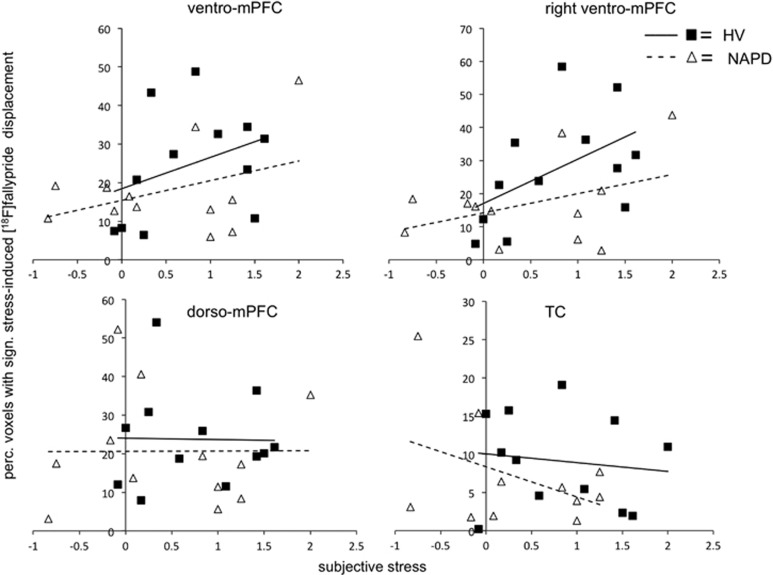
Association between the spatial extent of stress-induced [^18^]fallypride displacement and subjective stress in the whole sample. Subjective stress and the spatial extent of stress-induced radioligand displacement were associated in ventro-mPFC (trend, *P*=0.06) and, more specifically, right ventro-mPFC (*P*=0.02) in the whole sample, but not in dorso-mPFC (*P*=0.93) and TC (*P*=0.33). For visualization purposes, HV and NAPD were depicted separately. HV, healthy volunteer; mPFC, medial prefrontal cortex; NAPD, non-affective psychotic disorder; TC, temporal cortex.

In the whole sample, stress-induced radioligand displacement in mPFC (F(23)=0.11, *P*=0.74) or TC (F(23)=0.88, *P*=0.36) was not associated with subjective stress. The association between the spatial extent of stress-induced mPFC radioligand displacement and subjective stress in the whole sample did not reach significance (F(23)=1.71, *P*=0.2). When looking at mPFC subregions, the association between subjective stress and the spatial extent of stress-induced radioligand displacement in ventro-mPFC (F(23)=2.48, *P*=0.09) and dorso-mPFC (F(23)=0.15, *P*=0.87) was not significant (Figure 4). Further investigation revealed a significant positive association between subjective stress and the spatial extent of stress-induced radioligand displacement in right ventro-mPFC (F(23)=4, *P*=0.03; Figure 4), but not left ventro-mPFC (F(23)=0.83, *P*=0.45). Subjective stress was not associated with the spatial extent of stress-induced radioligand displacement in TC (F(23)=0.63, *P*=0.54; Figure 4).

The spatial extent of stress-induced radioligand displacement (*b*=1.13, *t*(7)=7.75, *P*=0.001), but not stress-induced radioligand displacement (*b*=−0.22, *t*(7)=−2, *P*=0.12), in ventro-mPFC was positively associated with duration of antipsychotics-free period.

Psychotic symptoms during the scan in NAPD were not associated with stress-induced radioligand displacement in mPFC (*b*=1.25, *t*(11)=−0.13, *P*=0.9) or TC (*b*=−1.42, *t*(11)=−0.67, *P*=0.53), or the spatial extent of stress-induced radioligand displacement in mPFC (*b*=−3.68, *t*(11)=0.51, *P*=0.62) or TC (*b*=−3.24, *t*(11)=−0.63, *P*=0.55). PANSS positive, negative or general symptoms in NAPD were also not associated with stress-induced radioligand displacement or the spatial extent of stress-induced radioligand displacement in mPFC or TC (Table 3).

Adding years off antipsychotics as a covariate did not change the results. Moreover, antipsychotics-naive NAPD and antipsychotics-free participants did not differ in stress-induced radioligand displacement or the spatial extent of stress-induced radioligand displacement in any of the identified regions (data not shown).

Finally, cumulative haloperidol equivalents (antipsychotics in the past) were not associated with stress-induced radioligand displacement in mPFC (*b*<0.01, *t*(11)=−0.47, *P*=0.65) or TC (*b*<0.01, *t*(11)=1.05, *P*=0.32), or the spatial extent of stress-induced tracer displacement in mPFC (*b*<−0.01, *t*(11)=−0.61, *P*=0.55) or TC (*b*<−0.01, *t*(11)=−0.37, *P*=0.72).

## Discussion

Using [^18^F]fallypride PET, the effect of psychosocial stress on extrastriatal DA signaling was investigated in HV and NAPD. In accordance with previous work, extrastriatal DA release^6^ and the spatial extent (area/size of DA release in voxels) of DA release^3, 31^ served as primary outcome measures of stress-related DA signaling. We showed that psychosocial stress increases extrastriatal DA signaling in HV: both DA release and the spatial extent of DA release increased in mPFC and TC. Moreover, we did not find evidence for altered stress-induced extrastriatal DA signaling in NAPD. This is based on the observations that (i) psychological stress increased DA signaling to a similar extent in HV and NAPD, (ii) subjective stress and the spatial extent of stress-induced DA release were similarly associated in HV and NAPD and (iii) stress-related DA signaling was not associated with positive, negative or general symptom scales of the PANSS in NAPD.^34^

BP_ND_ values in frontal and temporal areas were in ranges comparable to previous studies^6, 28^ although inter-individual variability was observed in the hippocampus and amygdala, which may be the result of the inherent small size of these structures. The observation that stress increased mPFC DA signaling in HV confirm previous data.^5, 6^ In addition, increases in DA signaling in TC were observed. Although stress-induced TC DA signaling in humans has not been reported before, it is consistent with functional magnetic resonance imaging studies using the MIST,^47, 48^ suggesting that these effects might be, in part, DAergic.

Contrary to expectations, differences in stress-induced frontal and temporal DA signaling between HV and NAPD were not observed. In combination with the absence of a correlation between measures of stress-induced DA signaling and psychotic symptoms (during scan or assessed with PANSS), these results could suggest that stress-related extrastriatal DA signaling is unaffected in NAPD. Here, we offer four explanations.

First, these results seemingly contrast with the hypothesis of DA-mediated hypofrontality in psychosis.^18, 19^ However, the concept of hypofrontality is often assessed indirectly (for example, cerebral blood flow) and in the context of cognitive performance,^18, 49, 50^ not stress. Little *in vivo* evidence exists for D_2/3_-mediated hypofrontality in psychotic disorder^10^ and a positive association between amphetamine-induced PFC DA release measured with [^18^F]fallpyride and schizotypal personality traits^29^ may even suggest increased cortical DA transmission in psychotic disorder. Although inconsistent,^51, 52, 53^ changes at the D_1_ receptor have been observed in schizophrenia. Moreover, experimental animal work suggests an important role for PFC D_1_ receptors in the stress response^54^ and a D_1_, but not D_2_, agonist can restore stress-related DAergic PFC–striatum interactions.^55^ Altogether, this could indicate that, although DA transmission at D_2/3_ during stress may be unaltered in psychotic disorder, activity at the D_1_ may be abnormal.

A second viable explanation may be that the absence of differences between HV and NAPD could be explained by the relatively low amount of acute psychotic symptoms (PANSS score; Table 1). This would, however, go against evidence that increased stress sensitivity is present in those at risk for psychotic disorder,^56^ non-acute psychotic disorder^57^ and even remitted psychotic disorder.^58^ In addition, stress-induced increases in psychotic symptoms during the scan confirmed increased stress sensitivity in our sample of NAPD. We recently reported a negative correlation between the spatial extent of mPFC DA release and subjective stress/subclinical psychotic symptoms in healthy first-degree relatives of individuals with psychotic disorder.^3^ This could suggest functional cortical DAergic alterations in the stress response in some, but not all, individuals across the psychosis continuum. One way to investigate whether stress-related PFC DA signaling is dependent on illness phase is the addition of a group of acutely psychotic NAPD.

A third explanation may be that the use of [^18^F]fallypride has contributed to the absence of group differences. Amphetamine-induced PFC DA release quantified with fallypride has mostly yielded negative results.^25,26,28^ However, three separate studies using the MIST^3, 6^ (including the current one), as well as a study investigating response inhibition,^59^ have reported PFC DAergic activity measured with fallypride. While the reason for this discrepancy between stimulant- and task-based studies using [^18^F]fallypride is unclear, it may be related to the different mechanisms of action task- and stimulant-induced DA release in the cortex. Whereas psychological tasks elicit increased DA synthesis and release, corresponding with increased cell firing,^60^ stimulants increase extracellular DA release through DA and noradrenaline transporter blockade^61^ and decrease overall cell firing.^62^ A replication study with higher affinity radioligands such as FLB 457 (refs. 26, 63) could be useful to assess the suitability of fallypride to detect task-induced cortical DA release, as has been done recently for stimulants.^26^

A final explanation could be that the sample displayed abnormalities in cortical neurotransmission not directly related to the DA system. This assumption is based on the observation that cognitive and negative symptoms in NAPD were not associated with stress-related DA signaling. One potential candidate neurotransmitter system could be glutamate. Glutamate transmission in the cortex has an essential role in stress processing,^64^ and cognitive and negative symptoms of schizophrenia have been associated with altered frontal glutamate activity,^65^ but not always consistently so.^66^ Thus, alterations in cortical glutamate transmission could potentially account for negative and cognitive symptoms in the sample of NAPD while also explaining their increased stress sensitivity to the task.

Although there may be multiple explanations for the absence of differences between NAPD and HV, stress-induced mPFC DA release^6^ and the spatial extent of mPFC DA release^3^ are associated with physiological and behavioral parameters. This suggests that PFC DAergic processing has a functional role in the stress response, which is potentially unaltered in NAPD. This was reflected in the correlation between the subjective stress response and spatial extent of ventro-mPFC DA release in the current study. However, an association between subjective stress and ventro-mPFC DA release was not observed. Although high correlations were observed between the spatial extent of DA release and DA release, this may indicate that increases in subjective stress are associated with a greater area of DA release without altering the amount of DA released. This could be interpreted as a compensatory processing mechanism, where increased resources are necessary to obtain the same result.

The spatial extent of ventro-mPFC DA release in response to stress increased as NAPD were longer off antipsychotics. Two possible explanations exist for this association. First, as NAPD are longer off antipsychotics, their DAergic stress response may progressively approximate that of HV. This is in line with an association between D_1_ receptor density and drug-free interval^67^ and could suggest that DA receptor density may normalize following prolonged exposure to antipsychotics. The association between time off antipsychotics and the spatial extent of stress-related DA release may reflect gradual homeostatic downregulation of PFC D_2/3_ receptors, previously upregulated through extended antipsychotics blockade, although such upregulations are dependent on mode of antipsychotics administration.^68, 69^

An alternative explanation may be that as acute psychotic symptoms decrease, DAergic abnormalities normalize. This is in line with work showing that striatal DA function of remitted schizophrenia patients^70^ and antipsychotics-treated schizophrenia patients^71^ is more similar to HV. However, this explanation goes against alterations in stress sensitivity that persist beyond acute psychotic disorder^58^ and the observation that the MIST increased psychotic symptoms in NAPD. Here, again, an acutely psychotic group of NAPD could be of added value.

### Strengths and limitations

The current findings need to be interpreted in light of strengths, limitations and sample size.

Strengths of the study include minimal past drug use in the sample, thereby excluding substance-induced NAPD and associated confounds in the DA system. Given that, in particular, cannabis use is associated with psychotic symptoms^72^ and DA function,^73, 74, 75^ this may have increased our sensitivity to investigate stress-related DA function. Moreover, the single infusion paradigm limited within-subjection variation, further decreasing measurement error. Finally, the direction and location of task effects in HV were similar to a previous study using an identical design, which suggest a degree of stability.^5^

Some limitations of the study need to be addressed. A general limitation is that the single infusion protocol with fallypride used in the current study has not been associated with measures directly related to DA activity, hence use of the term ‘DA signaling'. Moreover, striatal DA signaling could not be reliably investigated; actual and simulated data^31^ indicate that the current design would produce unreliable estimates for the striatum, given the slow time course of radioligand binding. Future [^18^F]fallypride studies could increase scan duration or, in the case of a single infusion paradigm, prolong the control condition to investigate striatal and extrastriatal DA signaling simultaneously.

Because of model assumptions and to limit stress exposure to the scanning period, the task order was fixed to control–experimental, similar to previous work.^5, 14^ Although this may have introduced order effects, a recent study demonstrated stress-induced DA release independent of the order of conditions.^6^ This makes it unlikely that order effects had a major effect on our outcome measures.

In addition, benzamide binding is affected by cerebral blood flow.^76^ However, in response to behavioral challenges^43^ and in low-binding areas,^77^ regional cerebral blood flow effects are rather small and are not expected to explain the presented results. Other studies with a single infusion paradigm have discussed this issue in greater detail.^5, 30, 33, 45^

In the absence of a task-induced effect on plasma cortisol levels, our results could reflect socially desirable behavior or increased effort in the stress condition. The association between subjective stress and the spatial extent of ventro-mPFC DA release does, however, suggest an effect of the stressor. This is also confirmed from by data from one HV who was scanned in a control–control sequence (data upon request); changes in subjective stress or [^18^F]fallypride displacement were not observed.

Rather, the absence of cortisol effects may be related to time of day; a significant association between sampling time and cortisol (nmol l^−1^) in HV was observed. Both the current study as well as another recent study who failed to find an effect of the MIST on cortisol levels^6^ collected PET data in the afternoon. In contrast, in a previous study, we did find an effect of the MIST on cortisol levels, but PET data were collected around noon. Future studies may, therefore, want to include physiological stress parameters that are less sensitive to time of day than cortisol.

Another observation was that MIST effects on the spatial extent of stress-induced ventro-mPFC DA release were smaller than previous work using an identical acquisition protocol (~25% here vs ~50%).^3^ This may be related to different versions of the task; the current study used an auto-adjust version (set to 70% correct responses), whereas a manually calibrated task (aiming at 90% correct response) was used previously. Task differences may have affected the perceived stressfulness of the paradigm and, correspondingly, DAergic processing. Moreover, image preprocessing software, scanner type and head fixation procedures may further explain these between-study differences.

Some limitations related to the sample also need to be addressed. Although NAPD were off antipsychotics for longer than 1 year, past antipsychotic use may have affected DA receptor density and thus masked subtle illness-related effects on stress-induced DA signaling. Although this is a limitation we acknowledge, repeating the analyses with time off antipsychotics as a covariate did not affect the results described in this manuscript. A sample of neuroleptic-naive participants could be valuable in detecting alterations in the extrastriatal DAergic stress response, if present, associated with NAPD. In addition, the NAPD sample included five individuals with brief psychotic disorder as their main diagnosis (Supplementary Table 1); low-grade residual symptoms in these individuals may have limited the power to detect associations between stress-related DA signaling and psychotic symptoms. Finally, *post hoc* power calculations indicated that group differences with effect sizes (Cohen's *d*) up until 0.5 may have been overlooked. To detect small-to-moderate group differences, replication with larger sample sizes is essential.

## Conclusions

Preclinical^16, 17^ and human^3^ studies have previously shown that stress affects DAergic activity in frontal cortical areas. The preliminary evidence presented here does not suggest altered extrastriatal DA signaling in the context of stress in NAPD. While we have demonstrated that frontal DA signaling is functionally relevant in the stress response, it is not clear how this is related to the putative link between stress and psychotic disorder. Follow-up studies in acutely psychotic and neuroleptic-naive NAPD could provide new insights into the role of stress-related extrastriatal DAergic processing in NAPD.

## Figures and Tables

**Table 1:** Sample demographics

	*HV*	*NAPD*	*Statistics (*P*-value, test statistic)*
Gender			(1, 0^a^)
Male	8	8	
Female	4	4	
Age	48.08 (9.94)	44.67 (11.24)	(0.44, −0.79^b^)
Education^c^	5.83 (1.4)	5.33 (1.44)	(0.4, 0.86^a^)
Smoking			(0.38, 0.54^a^)
Nonsmoker	11	10	
Smoker	1	2	
Cannabis lifetime^d^	0.23 (0.83)	0.67 (1.23)	(0.31, 1.04)
Other drugs lifetimed,^e^	0 (0)	0.01 (0.04)	(0.31, 1.04)
Injected radioligand (MBq)	189.83 (8.2)	187.92 (10.86)	(0.4, −0.85^b^)
Specific activity (GBq)	2611.42 (872.96)	2146.25 (1198.6)	(0.98, −0.03^b^)
Current symptoms^f^	—	11.83 (3.93)	—
Years off AP	—	7.09 (4.96)	—
Cumulative haloperidol equivalents^g^	—	4303.07 (12 280.64)	—

Abbreviations: AP, antipsychotics; HV, healthy volunteer; NAPD, non-affective psychotic disorder.

a Chi^2^ test.

b *T*-test.

c Highest finished education, scored on a scale ranging from 1 (primary school) to 8 (Masters degree).

d Lifetime use scored on a scale ranging from 1 (one to five times) to 8 (>100 times).

e Stimulants, sedatives, opiates, cocaine, psychedelics, XTC, MDMA, PCP and inhalants subscales.

f Positive subscale of the Positive and Negative Syndrome Subscale (PANSS).

g Cumulative haloperidol equivalents were calculated by converting the weekly antipsychotics dose to haloperidol equivalents and multiplying it by the number of weeks the antipsychotics were taken.

**Table 2:** Extrastriatal stress-induced [^18^F]fallypride displacement in HV and NAPD

*Region*	*HV stress-induced [*^*18*^*F]fallypride displacement* *mean Z(γ)*^a^ *(s.d.)*	*NAPD stress-induced [*^*18*^*F]fallypride displacement* *mean Z(γ)^a^* *(s.d.)*	P*-value group diff. Z(γ)*	T*-value group diff. Z(γ)*	*HV spatial extent of stress-induced [*^*18*^*F]fallypride displacement* *mean* n*%*^b^ *(s.d.)*	*NAPD spatial extent of stress-induced [*^*18*^*F]fallypride displacement* *mean* n*%^b^* *(s.d.)*	P*-value group diff. spatial extent*	T*-value group diff. spatial extent*
*Frontal lobe*								
mPFC^c^	2.68 (3.99)	1.59 (2.98)	0.46	−0.76	24.92 (12.16)	19.12 (10.67)	0.23	−1.24
								
*Temporal lobe*								
Temporal CTXc,d,^e^	12.88 (4.02)	13.79 (3.02)	0.54	3.21	8.96 (6.49)	7.34 (7.05)	0.27	−1.14
Hippocampus^d^	−0.42 (1.08)	−0.39 (0.74)	0.95	0.06	<1%	1.8 (3.1)	0.3	1.06
Parahippocampal gyrus^d^	−0.59 (82)	−0.72 (0.47)	0.65	−0.45	1.34 (1.83)	1.08 (1.18)	0.68	−0.42
Amygdala^d^	−0.17 (0.13)	−0.48 (0.83)	0.47	−0.74	2.07 (6.38)	<1%	0.43	−0.81

Abbreviations: CTX, cortex; diff., difference; HV, healthy volunteer; mPFC, medial prefrontal cortex; NAPD, non-affective psychotic disorder.

a Stress-induced [18F]fallypride displacement (*Z*(γ)).

b Percentage of total voxels in region of interest (ROI) showing significant stress-induced [18F]fallypride displacement (*Z*-value of γ).

c Significant stress-induced increase in tracer displacement and spatial extent of tracer displacement in HV and NAPD (*P*<0.05).

d One outlier removed with Cook's distance >4/12.

e Temporal cortex (inferior and superior temporal gyri).

**Table 3:** Associations between stress-induced [^18^F]fallypride displacement and psychotic symptoms on the Positive and Negative Syndrome Subscale (PANSS) in NAPD

	*Association between stress-induced [*^*18*^*F]fallypride displacement (Z(γ)) and PANSS symptoms*				*Association between spatial extent of stress-induced [*^*18*^*F]fallypride displacement (% voxels) and PANSS symptoms*			
	*Coefficient*	*95% CI*	T*-value*	P*-value*	*Coefficient*	*95% CI*	T*-value*	P*-value*
*Positive subscale*								
mPFC	0.16	−0.36 to 0.69	0.72	0.5	0.88	−1.09 to 2.84	1.03	0.33
Temporal CTX	−0.05	−0.68 to 0.56	−0.22	0.84	0.9	−0.42 to 2.21	1.57	0.16
								
*Negative subscale*								
mPFC	0.29	−0.95 to 1.53	0.54	0.61	−0.45	−5.26 to 4.37	−0.21	0.84
Temporal CTX	−0.7	−2.02 to 0.62	−1.22	0.26	0.49	−2.97 to 3.94	0.33	0.75
								
*General subscale*								
mPFC	0.05	−0.58 to 0.52	−0.12	0.91	0.26	−1.83 to 2.34	0.28	0.78
Temporal CTX	−0.16	−0.77 to 0.45	−0.6	0.57	0.48	−0.98 to 1.94	0.76	0.47

Abbreviations: CI, confidence interval; CTX, cortex; HV, healthy volunteer; mPFC, medial prefrontal cortex; NAPD, non-affective psychotic disorder.

## Notes

This work was supported by an ERC consolidator grant (ERC-2012-StG, project 309767—INTERACT) to IMG. DH was supported by a Maastricht University post-doc Kootstra fellowship, the Network of European Neuroscience schools (NENS) and South Limburg University fund (SWOL). DC was supported by a Maastricht University post-doc Kootstra fellowship. Data presented in this manuscript are available to collaborators upon request. The remaining authors declare no conflict of interest.
