# Supplementary material for: No evidence for attenuated stress-induced extrastriatal dopamine signaling in psychotic disorder
Source: Transl Psychiatry. 2015 Apr 14;5(4):e547–. doi: 10.1038/tp.2015.37 (PMC4462602; doi:10.1038/tp.2015.37)
Supplement: Supplementary Table 1 [file tp201537x1.doc]

**Supplemental Table 1** Diagnosis and medication characteristics of NA-PD

|  | **Gender** | **DSM-IV Psychotic Disorder Diagnosis** | **Past antipsychotic medication type** | **Past antidepressants**  **(yes/no)** | **Past benzodiazepines**  **(yes/no)** |
| --- | --- | --- | --- | --- | --- |
| **Subject #1** | F | Brief Psychotic Episode (298.8) | Haloperidol | - | - |
| **Subject #2** | M | Schizophrenia Paranoid Type (295.30) | Phenothiazine, haloperidol | - | - |
| **Subject #3** | M | Schizophrenia Paranoid Type (295.30) | Risperidone, pimozide | - | Yes (infrequently; not on day of scan) |
| **Subject #4** | F | Brief Psychotic Episode  (298.8) | Haloperidol, olanzapine | - | - |
| **Subject #5** | F | Brief Psychotic Episode  (298.8) | Olanzapine, pimozide | Yes (past) | Yes (past) |
| **Subject #6** | F | Schizophrenia Undifferentiated Type (295.9) | Risperidone, haloperidol, thioridazine, penfluridol | - | Yes (past) |
| **Subject #7** | M | Psychosis NOS (298.90) | Olanzapine | Yes (past) | Yes (past) |
| **Subject #8** | M | Psychosis NOS (298.90) | Sertindol | Yes (past) | Yes (past) |
| **Subject #9** | M | Brief Psychotic Episode  (298.8) | - | - | - |
| **Subject #10** | M | Brief Psychotic Episode  (298.8) | - | Yes (past) | - |
| **Subject #11** | M | Psychosis NOS (298.90) | - | - | - |
| **Subject #12** | M | Schizophrenia Paranoid Type 295.30 | - | Yes (past) | - |
